# Outcomes of Pain Management Training for the Fourth- and Fifth-Year Medical Students

**DOI:** 10.1155/2023/6080769

**Published:** 2023-08-25

**Authors:** Ayano Saeki, Yumiko Takao, Keiichiro Suzuki, Munetaka Hirose

**Affiliations:** ^1^Department of Anesthesiology and Pain Medicine, Hyogo Medical University, Faculty of Medicine, Nishinomiya, Hyogo, Japan; ^2^Department of Biochemistry, Hyogo Medical University, Faculty of Medicine, Nishinomiya, Hyogo, Japan

## Abstract

Pain management is a major medical issue. However, current medical education in Japan is inadequate with regard to training students to properly assess patients with acute and chronic pain and plan their treatment. Therefore, starting in 2019, Hyogo Medical University established a multidisciplinary educational system to better train medical students to provide pain care. The course, called clinical pain study, is offered to fourth- and fifth-year medical students. Fourth-year students learn the scientific aspects of pain through clinical practice. In this study, we assessed students' understanding of pain management based on the results of pretests and posttests performed before and after their practicum. These tests were administered from November 2019 to April 2022 to 263 fourth- and fifth-year medical students who took the clinical pain study class. The test results were compared in terms of the percentage of correct answers and the total score for each question using McNemar's chi-square test and paired *t*-tests, respectively. The results showed a significant improvement in the mean of the total score, confirming the improvement in medical students' knowledge (6.43 vs. 7.35 points; *p* < 0.001). Based on the results, overall, pain education at the university has had positive outcomes and will therefore be continued in the future.

## 1. Introduction

Pain management is part of the basis of patient care behavior, and relief from pain is the starting point of medical care. Physicians therefore need to develop and maintain skills in assessing and managing pain to provide appropriate care. Yet, such skills have been reported to be inadequate, despite being essential. Various pain education curricula have been developed and assessed to address this issue. Such curricula have been shown to achieve short-term improvements in knowledge, attitudes about care, and clinical competence. Watt-Watson et al., for example, developed and implemented a one-week, 20-hour pain education curriculum for six undergraduate health chemistry students at the University of Toronto; they reported significant improvements in pain knowledge and beliefs between preeducation and posteducation [1]. Sloan et al. similarly implemented a multimethod curriculum to educate third-year medical students about cancer pain [2]. Based on the results of cancer pain OSCEs administered 10–16 weeks after this training, the group that received education performed better than the control group, which did not receive education (*p* < 0.05). The students who received training also performed significantly better four months later than the control group.

In Japan, however, pain education has not been conducted consistently or adequately throughout the country, and pain is taught separately within each department and medical specialty as one of the symptoms of different diseases [3]. Despite the importance of pain relief for patients, few physicians in Japan are trained as pain management specialists. It is likely, then, that patients with pain often receive unsatisfactory pain management [4, 5]. Medical students are unable to connect knowledge and treatments based on the International Association for the Study of Pain's (IASP) classifications of pain (e.g., nociceptive pain, neuropathic pain, and nociplastic pain) and are unable to apply this knowledge to clinical practice after graduation [6–8]. To overcome this problem, Japan needs to implement more education on pain. Specifically, curricula specific to pain management need to be developed and implemented, and the outcomes need to be evaluated and analyzed. Pain education improves medical students' and healthcare professionals' understanding of pain mechanisms, assessment methods, and treatments. Practical experience, such as clinical practice and working with pain management teams in hospitals, is also essential. In addition to pain management training for students, it is also important to provide continuing education for professionals. Keeping doctors and healthcare professionals up-to-date with the latest knowledge and skills in pain management will improve their ability to assess and manage their patients' pain.

In 2019, the authors implemented a new system to better train medical students in pain management. The Pain Education Center, which was established in the same year, provides education on pain in collaboration with other professions. Here, the fifth-year medical students participate in the treatment of patients being treated for chronic pain and cancer pain at the Pain Clinic and Palliative Care Center. A newly established course, called clinical pain study, provides scientifically based pain education for fourth- and fifth-year medical students at the lead author's university, with the involvement of several medical professionals and researchers involved in pain management. The purpose of the present study was to objectively evaluate the effectiveness of the clinical pain study' course by testing the fourth- and fifth-year medical students' understanding of pain before and after the class.

## 2. Materials and Methods

### 2.1. Participants

This study was approved by the Ethics Review Board of Hyogo Medical University from 2019 to 2021 (approval no. 202208–006). The participants were fourth- and fifth-year medical students who took a course called clinical pain study. All personal information in the data was de-identified. Participation in the study was voluntary. A disclosure statement informed the students that their participation and scores in the study would not affect their grades.

### 2.2. Curriculum

**Figure 1:**
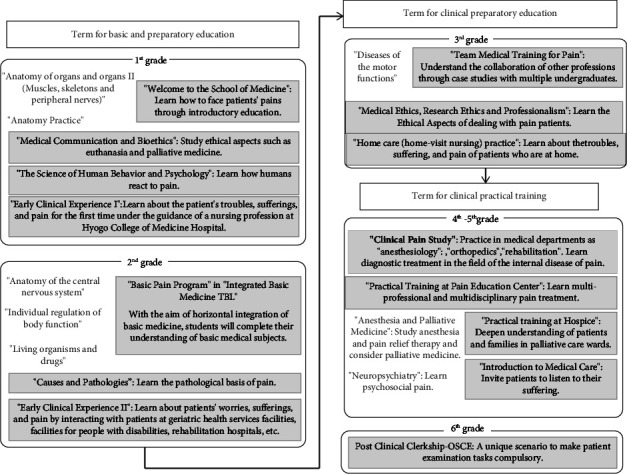
Outline of a new multidisciplinary educational curriculum started at Hyogo Medical University in 2019 to train students in pain treatment. TBL: team-based learning; OSCE: objective structured clinical examination.

To implement the plans described above, beginning in August 2019, we drastically changed the educational curriculum at Hyogo Medical University and increased the number of staff to provide lectures and practical training. In addition to the establishment of the Pain Education Center, the clinical pain study course, and the team medical training for pain, we started a program called early clinical experience (Figure 1). In this program, first- and second-year medical students observed actual medical practice and learned about the problems patients face with pain by interacting with them. In the training for the pain team, small teams were formed with third-year medical students; fourth-year students from the faculties of pharmacy, nursing, and rehabilitation; and clinical psychology students from other universities. They studied case studies of patients with pain to better understand clinical pain. In the fifth year of medical school, students received hospice training, called practical training at hospice, to learn about cancer pain. Medical students took the clinical pain study course in their fourth year before undergoing practical training. This included lectures by pain clinic doctors, orthopedic surgeons, clinical psychologists, basic researchers, and palliative care doctors on topics such as pain classification, the mechanisms of pain generation and maintenance, the brain's role in pain, pain treatment, pain psychology, musculoskeletal pain, and headaches. Practical training was carried out at the Pain Education Center, where the fourth- and fifth-year medical students were divided into groups of five to eight students for a full-day, weeklong practical training. During the practical training, students learned clinical skills, such as patient diagnosis, pain assessment, and pain management. Students also learned about treatment methods such as oral and regional anesthesia, as well as the psychological aspects of pain. In addition, during the practical training, students had the opportunity to assess patients' pain, observe fluoroscopic/ultrasound-guided nerve blocks performed by pain clinicians, and practice regional anesthesia on simulators.

### 2.3. Pretests and Posttests

**Figure 2:**
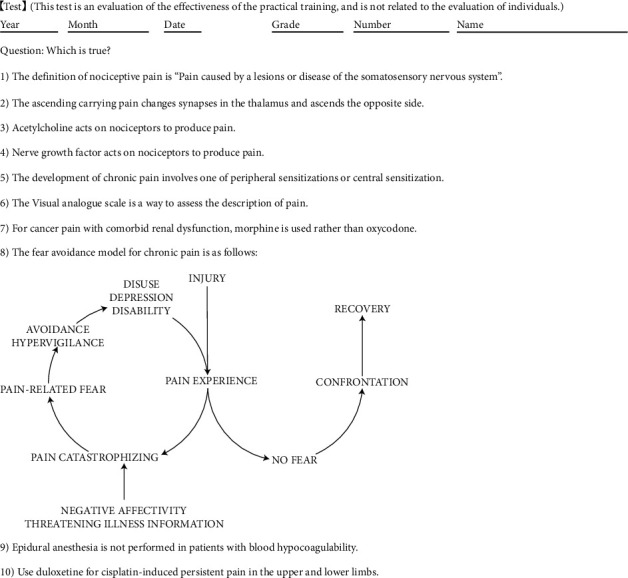
Details about the pretests and posttests conducted at the pain center.

Pretests and posttests were administered to assess the effectiveness of the curriculum. The tests consisted of 10 questions covering pain classification, physiological knowledge, evaluation methods, and treatment (Figure 2). Correct answers were scored as one point, and incorrect answers were scored as zero points, for a total score of 10 points. The pretest was administered on the morning of the first day of training for one week; and the posttest was administered on the morning of the fifth day, i.e., the last day of training. In the early evening of the fifth day after the posttest, a pain clinician asked questions and provided explanations on unclear points, focusing on test questions with the lowest percentage of correct answers; and the clinician also summarized the study.

### 2.4. Statistical Analysis

Data were analyzed using JMP Pro (v. 16.0.0; SAS Institute Inc., Cary, NC, United States). The pretest and posttest results were analyzed using McNemar's chi-square test and paired *t*-tests to compare the percentage of correct answers and the total score for each question, respectively. The significance was set at *p* < 0.05. Data collection and entry were performed by a clinical psychologist who was blinded to the study design.

## 3. Results and Discussion

### 3.1. Results

The study population included 284 fourth- and fifth-year medical students who took a course in clinical pain study. Nineteen medical students who had taken more than one practical course due to retention and two students who did not take the posttest because of absences were excluded from the study.

**Figure 3:**
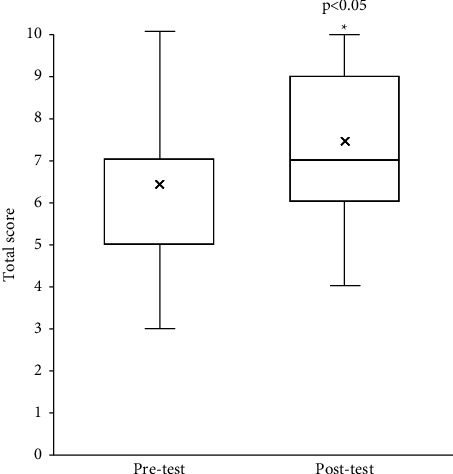
Total pretest and posttest scores. *Y*-axis represents total test scores (mean ± SEM). ^*∗*^indicates significance compared with the pretest (*p* < 0.05; paired *t*-test).

Table 1 shows the results for the percentage of correct responses on the pretest and posttests administered to the participants (*n* = 263) during the 2019–2021 school year, before and after their training at the Pain Education Center. Compared with the pretest, the posttest showed a significant increase in the percentage of correct responses to all questions, except Q3 and Q4.  Figure 3 shows the results for the total pretest and posttest scores. The posttest showed significantly higher scores than the pretest (6.43 ± 1.48 vs. 7.35 ± 1.63; *p* < 0.001).

## 4. Discussion

We developed and implemented a multidisciplinary educational system to train medical students in providing pain care. This involved collaboration with experts in diverse fields, including various departments involved in pain management and treatment. To continue this pain education curriculum, it is essential to train faculty with the necessary knowledge and skills [9]. In the present study, as a practical outcome, there was a significant improvement in the medical students' knowledge of pain.

Cooper et al. highlighted issues such as a lack of time among faculty, lack of funding for specialized education, and lack of interprofessional collaboration as obstacles to successful pain education [1]. Likewise, the specialists involved in our project had minimal time for student education since they devote most of their time to clinical research and patient care. Therefore, to continue this curriculum, it is necessary for faculty members to collaborate with each other. Meanwhile, pain education is still not offered at all universities in Japan. It is necessary, then, to make pain education widely available, even for those who are already physicians [10–12]. To improve this situation, with encouragement from the Ministry of Health, Labor, and Welfare, beginning in 2018, five collaborating universities established a common educational program on pain assessment and treatment. This included providing videos on pain education for medical students and doctors in Japan (https://manseino-itami.hosp.yamaguchi-u.ac.jp/program/index.html). We hope that such programs will be further promoted and disseminated to the public.

Our results showed no significant difference in knowledge improvement between Q3 and Q4 on the tests; and these questions concern whether acetylcholine/nerve growth factors act on nociceptors to produce pain. This is a basic medical science content area that some students have difficulty with. Since this content does not appear in many items on the national examination for doctors in Japan, it is possible that medical students are not adequately prepared for it. It is necessary to examine whether the content of our test was comprehensive in terms of pain knowledge and whether it was appropriate as an indicator of improved understanding. Demonstrating that short lectures and practical training can improve students' knowledge is an important and necessary part of examining the effectiveness of educational programs. We showed that short-term educational programs have the potential to improve medical students' knowledge of pain. Future research should therefore include assessments of the long-term retention of the content of such programs and their impact on patient management. Through our work, we aim to improve the management of patients' pain in Japan. Understanding the mechanisms of pain and learning strategies to holistically assess and respond to pain from a biopsychosocial perspective could lead to reductions in medical costs in Japan and ultimately improve many patients' quality of life [13].

### 4.1. Limitations

This study was based on test results taken by medical students at a single university over a two-year period. The sample was small. We confirmed improvements in knowledge and comprehension on tests administered as part of our clinical pain study course; we did not, however, determine whether the rate of correct answers on pain-related questions improved during graduation exams or on other tests. Although there have been many reports of short-term improvements in pain management skills following curriculum interventions, there are still few reports of the long-term maintenance of knowledge and skills, which should be examined in the future [6]. Although our test did not involve evaluation for advancement or other purposes, it is possible that its content may have been leaked to other groups. Lastly, although the program included team medicine with students from other professions, the test was limited to medical students only. Evaluations of other undergraduate groups are needed in the future.

## 5. Conclusion

We established a multidisciplinary educational program in 2019 to train medical students in treating pain. In the course, called clinical pain study, fourth-year medical students learned the scientific aspects of pain during clinical practice and attended lectures by various medical professionals involved in pain treatment. A comparison of pretest and posttest results for the fourth- and fifth-year medical students who took the course showed a significant improvement in the mean total score, confirming that students' knowledge was improved. We intend to continue this educational program and further develop it in postgraduate education to expand its role in patient-centered medicine.

## Figures and Tables

**Table 1:** Results for pretest and posttest correct response rate.

Questions	Correct answer	Pretest (%)	Posttest (%)
1	×	52.1	61.6^*∗*^
2	×	79.5	89.0^*∗*^
3	×	65.8	69.2
4	○	36.9	36.1
5	×	61.6	68.8^*∗*^
6	×	73.8	82.9^*∗*^
7	×	75.3	86.3^*∗*^
8	○	90.5	96.6^*∗*^
9	○	50.2	73.8^*∗*^
10	○	58.2	70.3^*∗*^

^*∗*^indicates significance compared with the pretest (*p* < 0.05; paired *t*-test).

## Data Availability

Data for the pretests and posttests are restricted by the institutional review board at Hyogo Medical University to protect students' privacy. Data are available from Ayano Saeki (ay-tsuji@hyo-med.ac.jp) for researchers who meet the criteria for access to confidential data.
